# Chronic Q Fever with No Elevation of Inflammatory Markers: A Case Report

**DOI:** 10.1155/2012/249705

**Published:** 2012-06-26

**Authors:** Matteo Boattini, André Almeida, Rita Barata Moura, João Abreu, Ana Sofia Santos, Miguel Toscano Rico

**Affiliations:** ^1^Department of Internal Medicine, St. Marta's Hospital, 1169-024 Lisbon, Portugal; ^2^Department of Cardiology, St. Marta's Hospital, 1169-024 Lisbon, Portugal; ^3^Centre for Vectors and Infectious Disease Research Doutor Francisco Cambournac, National Institute of Health Dr. Ricardo Jorge, Aguas de Moura, 2965-575 Setubal, Portugal

## Abstract

We describe the case of a 55-year-old man with a biological prosthetic aortic valve who suffered from epigastrium and right hypochondrium pain associated with intermittent night sweats. Liver biopsy showed infectious hepatitis pattern without pathognomonic features. *Coxiella burnetii* serology was suggestive of chronic Q fever, and modified Duke's criteria for endocarditis were also fulfilled. The authors present a brief literature review concerning chronic Q fever, emphasizing absent previous reports of chronic Q fever with hepatitis and endocarditis and no increase in inflammatory markers.

## 1. Introduction

Q fever is a worldwide zoonosis caused by the “rare and fastidious bacteria” [1] *Coxiella burnetii* (*C. burnetii*). Its incidence is unknown and may be underestimated [2]. Clinical and laboratory presentation can be polymorphic, nonspecific and represents a great challenge for clinicians. Left untreated chronic Q fever is a potential lethal condition [1].

## 2. Case Presentation

A 55-year-old man, lawyer, was admitted to the hospital because of abdominal pain located in the epigastrium and right hypochondrium, intermittent night sweats, and 3 kg involuntary weight loss over the preceding 8 months. Approximately 5 years earlier severe aortic valve stenosis, with left ventricular hypertrophy, paroxysmal atrial fibrillation, and heart failure (class III NYHA) were diagnosed, and aortic valve replacement was performed using a biological prosthetic valve. Furthermore, the patient was an alcohol user (70 g/day) and had endoscopically diagnosed chronic antral gastritis. He lived in an urban area but, approximately 18 months before admission, he traveled to Nepal living in settings with poor hygienic standards. Daily medications included omeprazole 20 mg.

Approximately one year before admission, the patient began to have intermittent, profuse night sweats associated with abdominal pain located in the epigastrium, and right hypochondrium. He reported no fever or chills. No remarks on routine physical examination performed by his attending physician were made, and Table 1 summarizes the results of prescribed laboratory tests. Serologies for human immunodeficiency virus (HIV), hepatitis B virus (HBV), hepatitis C virus (HCV), and VDRL were negative. Serologies for cytomegalovirus (CMV) and Epstein Barr virus (EBV) showed past infections. Two blood cultures were negative. A transthoracic echocardiography detected hemodynamically well-functioning aortic valve prosthesis, ascendant aortic dilatation, and no valve vegetations. An abdominal ultrasound revealed a biliary cyst of the liver. His physician eventually decided to prescribe sertraline for depression.

Four months before admission, the patient was still complaining of abdominal pain, with the same characteristics and was referred to our out-patient clinic. Laboratory tests were repeated (Table 1), protein electrophoresis was performed, and results were within normal range.

**Figure 1:**
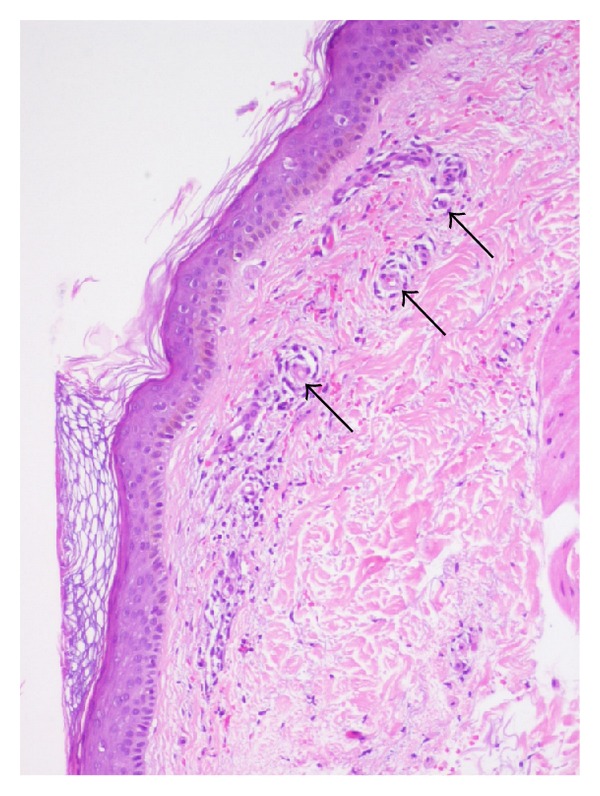
Skin biopsy. Perivascular dermatitis with purpura (arrows).

Meanwhile, during a work trip to Brazil, the patient developed lower limbs leukocytoclastic vasculitis (Figure 1).

Due to worsening of intermittent night sweats, persistence of abdominal pain, and 3 kg involuntary weight loss over the preceding 8 months with no fever, the patient was admitted to the hospital.

On examination, the patient was in good condition, the temperature was 37.1°C, the pulse rate 75 beats per minute, the blood pressure was 130/75 mmHg, and respiratory rate was 15 per minute. The oxygen saturation in ambient air was 98%, good oral and dental health. There was no superficial lymphadenopathy. There was no jugular venous distension. Auscultation of the heart revealed a mechanical second heart sound, a grade II/VI systolic murmur in the aortic area. No chest rales were detected. There were no conjunctival hemorrhages, Osler's nodes, Janeway's lesions, Roth's spots, splinter hemorrhages, or peripheral edema.

The electrocardiogram showed a normal sinus rhythm with nonspecific repolarization abnormalities.

**Figure 2:**
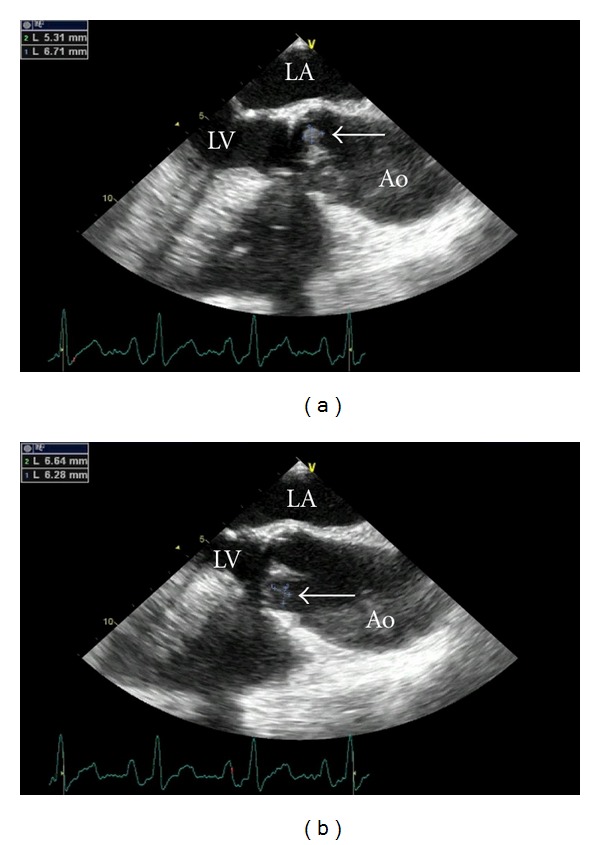
Transesophageal echocardiography. Echodense images on the aortic cups indicated by arrows. Left atrium (LA), left ventricle (LV), aorta (Ao).

Routine laboratory tests (Table 1) were performed and revealed hemoglobin 12.8 g/L [13.5–17.5], platelet count 139,000 per mm^3^ [150,000–450,000], aspartate aminotransferase (AST) 61 U/L [<50 U/L], alanine aminotransferase (ALT) 73 [<50 U/L], alkaline phosphatase (ALP) 110 [<120 U/L], ferritin 418 ng/mL [24–336], C-reactive protein (CRP) 4.1 [<5.0 mg/L], erythrocyte sedimentation rate (ESR) 15 mm/hr [<20], and rheumatoid factor (RF) 318 U/mL [<14]. Electrolyte panel and urinalyses were normal. Serologies for HIV, HBV, HCV, *Schistosoma*, *Borrelia*, *Rickettsia, Leishmania,* and VDRL were negative. Serologies for CMV, EBV, and *Toxoplasmose* showed past infections. Widal and Huddlesson reactions and research of *Plasmodium *sppwere negative as well. Two sets of blood culture specimens exhibited no growth. Protein electrophoresis showed IgG-K/*λ* monoclonal hypergammaglobulinemia. Abdominal ultrasound detected mild hepatosplenomegaly. Thoracic-abdominal-pelvic computer tomography disclosed gastric wall calcification and confirmed hepatosplenomegaly. To rule out endocarditis, transesophageal echocardiography was performed. Two very small echodense images suggestive of surgery-related alterations *versus* fibrosis were detected on the aortic cups (Figures 2(a) and 2(b)). Fundus examination showed no retinal abnormalities.

**Figure 3:**
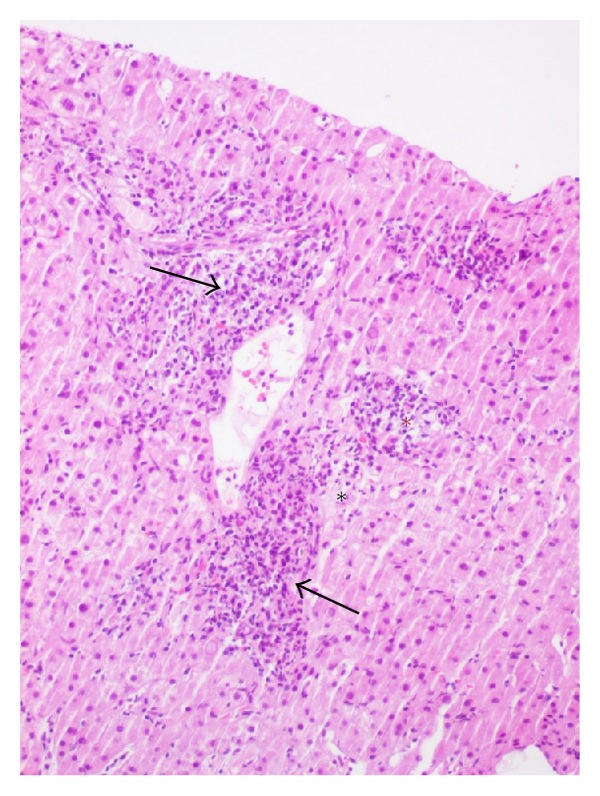
Liver biopsy. polymorphic inflammatory infiltrate of the portal spaces with extravasation into the hepatic parenchyma (black arrows), cell aggregates outlining granulomas (red star), focal necrosis of hepatocytes (black star).

Liver biopsy revealed infectious hepatitis without pathognomonic features (Figure 3) and with no fibrin ring granulomas described in Q fever. 

EDTA-whole blood and paraffin-included liver biopsy were then sent to CEVDI/INSA for *C. burnetii* laboratory diagnosis. The serologic evaluation was performed by Indirect Immunofluorescence Assay (IFA) using the commercial *C. burnetii *I+II IgG/A/M Immunofluorescence kit (Vircell Microbiologists, Granada, Spain). It revealed a high anti-phase I IgG with an end-point titer of 204,800 suggestive of chronic Q fever (anti-phase I IgG ≥800 was the reference cut-off, Table 2 for results). *C. burnetii *active infection was also confirmed by molecular testing. For this purpose, genomic DNA was extracted from buffy coat and liver tissue using DNeasy blood & tissue kit (Qiagen GmbH, Hilden, Germany). The presence of *C. burnetii *DNA was screened by polymerase chain reaction (PCR) in a nested reaction using the primers pairs Trans1-Trans2 and Trans3-Trans4 that target a transposon-like repetitive region in the agents genome [3]. Specific DNA was detected in liver tissue but not in blood, and *C. burnetii* identity was confirmed by amplicon sequencing. The quality of DNA in both samples was previously verified by amplification of human *β*-actin gene as internal control. Moreover, the absence of bacteria in blood sample was reinforced by a negative result also obtained for the isolation attempt. In fact, an additional buffy coat aliquot was inoculated in DH82 cells, routinely used in CEVDI for *C. burnetii* isolation, and after a standard 60-day incubation at 37°C and 5% CO_2_ atmosphere in BSL3 condition, no bacteria growth could be detected. The paraffin processing of the liver sample limited its utility for agents isolation.

Final diagnosis of chronic Q fever was made, with histologically documented infectious hepatitis and endocarditis by modified Duke's criteria [4]. Our patient met one of the major criterion of Q fever endocarditis (*C. burnetii* serology with anti-phase I IgG titer >800) and three minor criteria (predisposing heart condition with biological prosthetic aortic valve, immunological phenomena with RF positive, vascular phenomena with leukocytoclastic vasculitis). Furthermore, we probably may consider the valvular alterations detected by transesophageal echocardiography as a minor criterion (echocardiographic findings not meeting a major criterion).

Antimicrobial therapy with doxycycline 100 mg *bid,* and hydroxychloroquine 200 mg *tid* was started and the patient became asymptomatic within 8 days. 

The patient is still being followed in our out-patient clinic and he is adhering to treatment. He remains well and his weight gradually returned to normal (75 kg-Bode Mass Index 23,1), 8 months after the diagnosis. The titers of antibodies to *C. burnetii *have been falling in response to therapy. Results of the periodical serological tests are summarized in Table 2, together with results of the laboratory tests (Table 1).

## 3. Discussion

*C. burnetii* is a short and pleomorphic, strictly intracellular, gram negative coccobacillus with high resistance to chemical and physical agents as well as to antimicrobial therapy [5]. Humans are exposed to the disease through contact with animal hosts, such as, humans, ruminants (cattle, sheep, goats), pets, and rarely wild animals. Infection is manly acquired by the respiratory or digestive route [6].

Q fever may be acute or chronic. Acute infection can cause symptoms ranging from a flu-like illness to severe presentations like pneumonia and hepatitis. Only 1 to 5% of patients progress to chronic infection. This may develop insidiously months to years after the acute disease and the diagnosis is often delayed 12 to 24 months [7]. The most frequent and serious chronic presentation of Q fever is endocarditis. Less commonly, the disease may cause osteoarticular infections, vascular infections including infection of grafts, granulomatous hepatitis and chronic hepatitis, chronic pulmonary infection, and infection during pregnancy [5]. Chronic Q fever most often affects adult males, professionally exposed, with preexistent cardiovascular disease (valve lesion, valvular prosthesis, vascular graft), immunocompromised, living in rural areas, or who had shorts exposures, such as, farm visits or travels [2]. It should be considered for all patients with a heart valve lesion who present fever and negative blood cultures [1].

We believe that our patient could have been infected in Nepal although *C. burnetii* is endemic in Portugal as well. The patient reported that he had contacts with farm animals and he most likely consumed unpasteurized dairy products. 

Clinical manifestations of chronic Q fever vary widely, with low-grade fever and acute heart failure as the most common observed signs. Constitutional symptoms including anorexia, weakness, malaise, fatigue, anorexia, as in our patient, night sweats, and weight loss can also be present. Hepatosplenomegaly and purpuric rash due to circulating immune complexes are described as well [8]. Laboratory manifestations are typical of the cell-mediated inflammatory response [9]. Hematological abnormalities include mild elevation of both transaminases and alkaline phosphatase. Elevated ESR and hypergammaglobulinemy are seen in most patients, with significant increase in those diagnosed at a mean of 18 months [10]. Our patient suffered from a rare case of chronic Q fever with endocarditis and hepatitis with no increased inflammatory markers. 

Serology is the most used diagnostic method for Q fever. During acute infection, antibodies to phase II antigens are detected first, whereas persisting high levels of antibodies to phase I antigens are indicative of chronic Q fever infection. Chronic Q fever is suspected if titers of phase I IgG antibodies are >800 [2]. PCR assay is a method that could be used for the diagnosis of chronic Q fever but its efficiency is reduced in the serum samples due to small amount of bacterial DNA and elevated *C. burnetii* antibodies titer present [11]. Other diagnostic techniques include demonstration of *C. burnetii* in the tissues or isolation of *C. burnetii* in cells culture. 

Our patient's liver biopsy showed no fibrin-ring granulomas. This finding is consistent with literature because this type of granulomas has never been observed in the liver of patients with endocarditis [12].

Echocardiography, even if transesophageal, has significant limitations in diagnosing Q fever endocarditis due to the small size and nodular shape of the vegetations. Valvular abnormalities more frequently described are fibrosis and calcification [13]. In our case, valvular findings visualized by transesophageal echocardiography were not considered, at the beginning, suggestive of endocarditis vegetations. 

The combination of doxycycline with hydroxychloroquine for at least 18 months is the more effective therapy for chronic Q fever [8, 14].

Surgical valve replacement is generally indicated for hemodynamic reasons after at least 3 weeks of antimicrobial treatment [15].

Apyrexia within 7 days is the clinical expected response. Hepatomegaly and splenomegaly disappear within 2 to 12 weeks, and biochemical tests slowly return to normal.

During therapy serological testing and transesophageal echocardiography should be performed because of the possibility of later relapse, together with ophthalmologic examination aimed at monitoring toxicity from hydroxychloroquine [6].

In conclusion, we described a case of chronic Q fever with hepatitis and endocarditis that should help clinicians to consider this diagnosis, performing systematically *C. burnetii* serology, for all patients with heart valve lesions who present with nonspecific symptoms, even if they are apyretic and exhibit no elevation of inflammatory markers. 

## Figures and Tables

**Table 1:** Laboratory tests.

	Normal range	1 year before admission	4 months before admission	On admission	After 1 month of treatment
Hemoglobin (g/dL)	13.5–17.5	**14.4**	**14**	**12.8**	**13.7**
MCV (fL)	78–96	**82.5**	**82.3**	**78.8**	**83.4**
MCH (pg)	26–33	**27.6**	**27.7**	**25.7**	**27.5**
White-cell count (per mm^3^)	4,500–11,000	**7,380**	**7,210**	**9,090**	**7,100**
Platelet count (per mm^3^)	150,000–450,000	**220,000**	**185,000**	**139,000**	**177,000**
ALP (U/L)	30–120	**112**	**106**	**110**	**103**
AST (U/L)	<50	**48**	**43**	**61**	**31**
ALT (U/L)	<50	**53**	**36**	**73**	**35**
LDH (U/L)	<240		**206**	**234**	**218**
ESR (mm/h)	<20	**13**		**15**	**2**
CRP (mg/L)	<5.0	**2.2**	**5.0**	**4.1**	**3.2**
Ferritin (ng/mL)	24–336	**298**		**418**	**193**

**Table 2:** *C. burnetii *titers (IFA).

	On admission	After 1 month of treatment	After 2 months of treatment	After 6 months of treatment
Phase I IgM	200	50	50	<50
Phase I IgA	800	100	100	100
Phase I IgG	204,800	204,800	102,400	102,400
Phase II IgM	200	50	50	<50
Phase II IgA	<50	<50	<50	<50
Phase II IgG	204,800	409,600	204,800	51,200
